# Supplementary material for: Photon bubble turbulence in cold atom gases
Source: Nat Commun. 2021 May 28;12:3240. doi: 10.1038/s41467-021-23493-2 (PMC8163808; doi:10.1038/s41467-021-23493-2)
Supplement: Supplementary file 1 — Supplementary Information [file 41467_2021_23493_MOESM1_ESM.pdf]

Supplementary Information to: Photon Bubble Turbulence in Cold Atom Gases

R. Giampaoli,[1] J.D. Rodrigues *,[2,1] J.A. Rodrigues,[1,3] and J.T. Mendonça[1]

[1]*Instituto de Plasmas e Fusão Nuclear, Instituto Superior Técnico, Universidade de Lisboa, 1049-001 Lisbon, Portugal*

[2]*Physics Department, Blackett Laboratory, Imperial College London, Prince Consort Road, SW7 2AZ, United Kingdom*

[3]*Departamento de Física, Universidade do Algarve, Campus de Gambelas, 8005-139 Faro, Portugal*

Contents

I. Imaging 1

A. Imaging working principle 1

B. Imaging setup 3

C. Pump-probe calibration 4

II. Diffusion Approximation 5

A. Effective optical density 5

B. Statistics of photon scattering events 7

III. Finite-size photon bubble model 8

References 9

I. IMAGING

A. Imaging working principle

All the experimental results shown in this work have been obtained by means of the novel imaging technique developed particularly for this investigation and which we shall now describe in further detail. Typical absorption imaging techniques recover the 2D map of the full line-of-sight integrated atom density. In the case of 3D atom samples, important information about the sample density structure can be lost due to this integration effect. The technique developed here circumvents this limitation or, more precisely, it works such that line-of-sight integration effects are limited to small length scales, much smaller than the total system size.

In order to direct measure a quasi-2D density distribution, we first prepare the system such that the probe laser is absorbed just by a thin layer of atoms. This "priming" step is performed with a conveniently spatially-shaped pump beam, which is used to induce a ground-state change in the outer-lobes of atom cloud. The subsequent probe then selectively images a single thin atom slab. Due to the typical temperatures of about

* Corresponding author: j.marques-rodrigues@imperial.ac.uk

200 µK atom motion, which happens on the millisecond timescale, is essentially frozen during the entire imaging sequence, which happens on the microsecond timescale. The pump-probe procedure is depicted in Supplementary Figure 1:

- **a)** – Atom cloud immediately after the magneto-optical trap has been released. All atoms are in the $F = 3$ hyperfine state (the excited state lifetime is about 27 ns).
- **b)** – Pump stage: The pump beam is transversely carved by means of a thin metallic rod and brings the atoms located on the outer-lobes of the cloud to the $F' = 3$ excited state.
- **c)** – At the end of the pumping stage all atoms located at the outer lobes have decayed to $F = 2$ ground-state. The only atoms left at $F = 3$ are those located on a thin quasi-2D layer passing through the center of the cloud. Note that multiple excitation cycles may be necessary to induce the desired ground-state change. We make sure that the pumping step lasts long enough such that most of these atoms transition into the $F = 2$ ground-state - check Section I C.
- **d)** – The probe beam images the quasi-2D atom layer. To maximize the absorption signal the probe beam is kept at resonance with the electronic transition and its intensity kept low with respect to saturation, maintaining the linear absorption regime. Line-of-sight integration effects become only relevant at scales smaller than the thickness of the slab, around 230 µm.

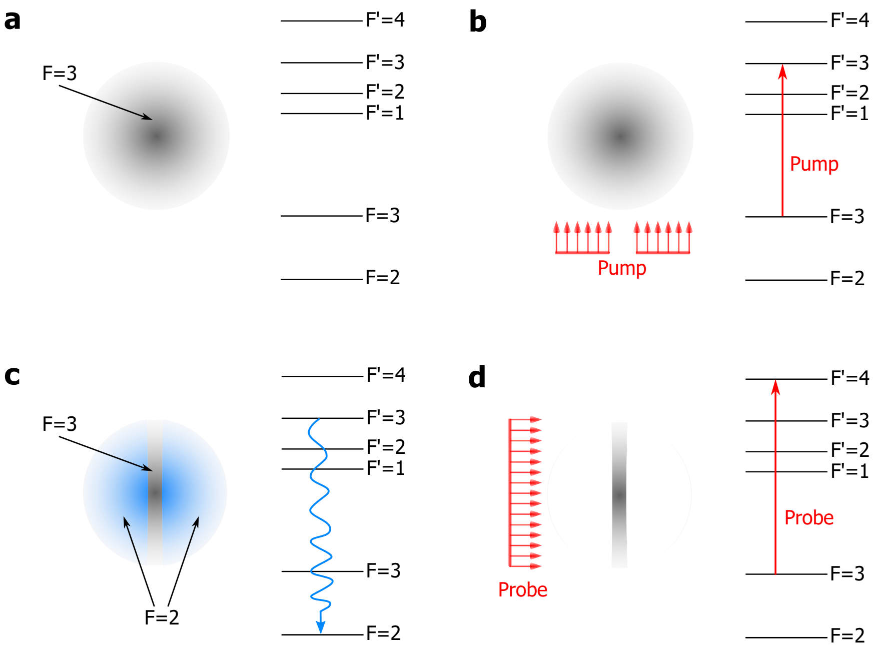

Supplementary Figure 1: Experimental imaging sequence. The different steps are described in the text.

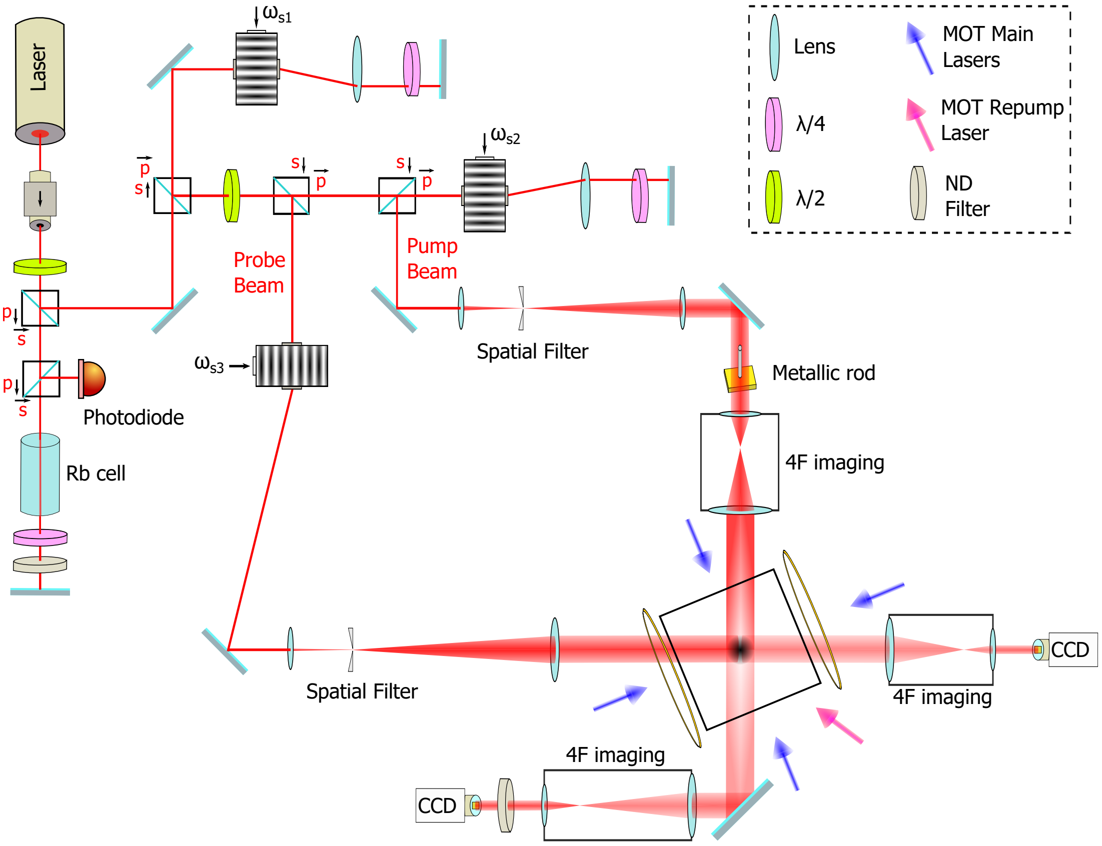

Supplementary Figure 2: Imaging setup. The details of the setup are described in the text.

B. Imaging setup

The full imaging setup is sketched in Supplementary Figure 2. The same laser diode (Sacher Lasertechnik Cheetah DFB-780) has been used for both pump and probe beams. A Saturated Absorption Spectroscopy (SAS) allows us to resolve the hyperfine structure of Rb^{85}. The SAS signal, is used together with a PID to lock the laser frequency at the crossover peak beetween the $F' = 2$ and $F' = 4$ transition, located approximately 90 MHz below the cycling $F = 3 \to F' = 4$ transition. Thereafter, the laser is sent through an acousto-optic modulator (AOM) in double passage configuration: this stage up-shifts the laser frequency onto the blue side of the cooling transition and allows a fine tuning of both pump and probe frequencies. The laser beam is then split in two branches, namely the pump beam and the probe beam. This task is performed by means of a half wave plate and a polarised beam-splitter, enabling the precise control of power balance between the two arms.

The probe power has been carefully optimised to be high enough to provide a good absorption signal but low enough not to saturate the electronic transition and maintain the linear absorption regime. The probe is then sent through an AOM in single passage. This is used both as a beam switch and to bring the probe frequency into resonance with the $F = 3 \to F' = 4$ transition. This beam is magnified with a telescope and cleaned up with a spatial filter in order to have a nearly-uniform intensity on the atom cloud. The probed beam is finally imaged onto a monochromatic CCD by a 4F imaging system.

The pump beam first goes through a second AOM in double passage configuration which works as a beam switch and brings the laser frequency into resonance with the $F = 3 \rightarrow F' = 3$ transition. After magnification and spatial filtering stages similar to the ones used for the probe beam, the pump is sent through a metallic rod ($\simeq$ 70 µm) mounted on a xyz translational stage. The rod is imaged onto the atom cloud by a 4F imaging system with numerical aperture on the atom cloud side of 0.045 and a magnification factor of 3.3. A small numerical aperture is essential in here to make sure that the imaged rod stays focused through the whole atom cloud. Finally, a CCD coupled to a 4F imaging system similar to the one used for the probe beam is used to properly center and focus the rod during the alignment procedure.

C. Pump-probe calibration

The pumping step must be long enough to ensure all atoms in the outer lobes of the sample have gone through the ground-state change described above. However, very long pumping times are no desirable as well, as this may lead to significant atom displacement during the entire pump-probe imaging sequence.

We performed several pump-probe experiments at different values of the pumping duration. In each run we measured the optical thickness of the atom cloud, with the results depicted in Supplementary Figure 3. The "with rod" series has been carried out with the pump beam transversely carved, thus maintaining the atoms located at the central quasi-2D layer in the shadow. These remain in the $F = 3$. The "without rod" series, on the other hand, has been measured without the rod induced shadow, i.e. pumping all the atoms in the sample into the $F = 2$ ground-state.

After few tens of microseconds, both optical density curves reach a plateau. The "with rod" set saturates at an higher optical density, which is the optical density of the thin quasi-2D atom slab. In our experiments we opted to pump the system for 70 µs, deep into the plateau regime, yet much shorter than the atom motion timescales.

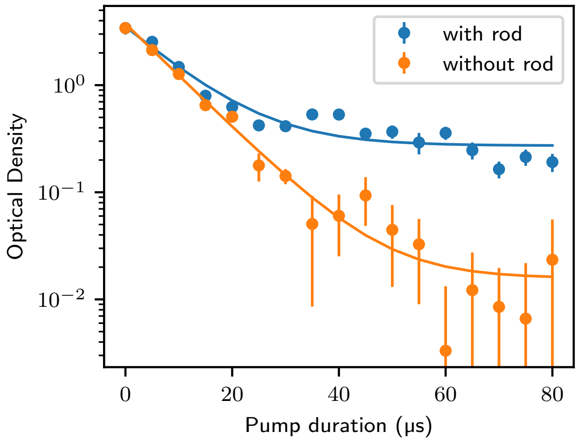

Supplementary Figure 3: Calibration of the pump duration. The optical density of the atom cloud, under the $F = 3 \to F' = 4$ transition, is plotted as a function of pumping duration in two different cases depending on the pump beam profile: a Gaussian beam ("without rod") and the same beam but transversely carved ("with rod"). The optical density here is a result of averaging over several CCD pixels and the error bar represents the respective standard deviation.

II. DIFFUSION APPROXIMATION

In this section, we show evidence of the diffusive character of photon transport in the turbulent regime. We begin by describing experimental measurements of the optical density in section A. Based on these measurements, in section B we report relatively simple Monte-Carlo simulations describing the full statistics of photon scattering events.

A. Effective optical density

In this section, we describe careful measurements of the optical density across the entire range of parameters while taking into consideration the effects coming from the finite linewidth of the lasers.

We define the transmission coefficient of a purely monochromatic laser as the ratio of the probe laser intensities after and before being partially absorbed by the atom cloud: $T(\delta_{\rm p};\delta_{\rm MOT}) = I(\delta_{\rm p};\delta_{\rm MOT})/I_0$, where $\delta_{\rm p}$ is the probe laser detuning (with respect to the electronic resonance), $\delta_{\rm MOT}$ is the MOT main lasers detuning at which the atom cloud is produced. In low saturation conditions, the single-frequency optical density is defined through the Lambert-Beer law as:

$$b(\delta_{\rm p};\delta_{\rm MOT}) \equiv -\ln\Big(T(\delta_{\rm p};\delta_{\rm MOT})\Big) = \ln\left(\frac{I_0}{I(\delta_{\rm p};\delta_{\rm MOT})}\right) = \frac{\sigma_0 \displaystyle\int_0^{\rm L} n(x,y,z)\,{\rm d}z}{1+4\left(\frac{\delta_{\rm p}}{\Gamma}\right)^2}, \tag{1}$$

where Γ is the transition linewidth, L is the length of the probed medium and σ_0 is the absorption cross section at resonance. For a finite-linewidth probe laser, the effective transmission coefficient is given by the

convolution of the single-frequency transmission coefficient $T_L(\delta_\mathrm{p};\delta_\mathrm{MOT})$ with the the laser spectrum $\mathcal{L}(\delta_\mathrm{p})$:

$$T^\mathrm{eff}(\delta_\mathrm{p};\delta_\mathrm{MOT}) = \int_{-\infty}^{+\infty} T(\delta_\mathrm{p}-\delta_\mathrm{p}^{'};\delta_\mathrm{MOT})\mathcal{L}(\delta_\mathrm{p}^{'})\,\mathrm{d}\delta_\mathrm{p}^{'}. \tag{2}$$

Consequently, we define the effective optical density $b^\mathrm{eff}(\delta_\mathrm{p};\delta_\mathrm{MOT}) = -\ln\Big(T^\mathrm{eff}(\delta_\mathrm{p};\delta_\mathrm{MOT})\Big)$. The single-frequency optical density at resonance, $b(0,\delta_\mathrm{MOT})$, can be accessed by measuring the transmission coefficient $T^\mathrm{eff}(\delta_\mathrm{p};\delta_\mathrm{MOT})$ over a large range of probe detunings and fitting the results to the expression in Eq. 2.

Using this method, we measured, for instance, $b(0,-3.5\Gamma) = 21$. Although viable in the stable regime, the above procedure is unpractical when applied in the turbulent regime, but it can be used to conveniently calibrate the pump-probe measurements by scaling the optical density of the slab to the optical density of the whole MOT. The scaling coefficient has been obtained as the ratio of the whole cloud optical density, measured in the stable regime, and the maximum optical density measured with the pump-probe at the same δ_MOT. This scaling coefficient is essentially determined by geometrical factors, which do not appreciably change when we range δ_MOT. From these data we can calculate $b^\mathrm{eff}(\delta_\mathrm{MOT};\delta_\mathrm{MOT})$, the effective optical density at the frequency of the MOT lasers. This operation can be performed by using the Eq. 2 to convolute the MOT main laser optical spectrum with the transmission spectrum built on the experimental values of $b(0,\delta_\mathrm{MOT})$. Results are shown in Supplementary Figure 4. We can observe a strong increase of the optical density at the MOT laser frequency as it is brought close to resonance. Moreover, this increase matches with the onset of the turbulent regime, demonstrating that it coincides with the increasingly stronger diffusive character of photon transport.

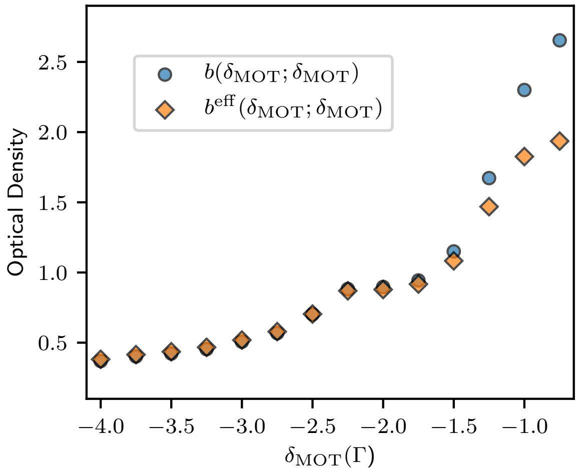

Supplementary Figure 4: Optical density seen by MOT main driving lasers. The increasing optical density approaching resonance demonstrates the transition into a regime dominated by diffusive photon transport, which coincides with the onset of the turbulent regime.

B. Statistics of photon scattering events

Photon scattering in driven cold atoms is a very intricate problem [1–4] that has yet to be fully described in a complete and systematic framework. In this section, we are going to describe a very simplified model of photon scattering and, based on the optical density measurements described before, perform Monte-Carlo simulations in order to retrieve the full photon scattering statistics. In our model, we consider only the inelastic scattering channel. We will also approximate emission by a simple Lorentzian curve centered around the driving laser frequency, thus ignoring the Mollow triplet. The frequency-dependent absorption is computed as $A(\delta; \delta_{\mathrm{MOT}}) = 1 - T(\delta; \delta_{\mathrm{MOT}})$, where $T(0; \delta_{\mathrm{MOT}})$ is determined by the optical density measurements above. Examples of emission and absorption spectra are shown in Supplementary Figure 5. The simulation is conducted by following many stochastic realizations of the scattering processes undergone by a single photon. For each MOT driving laser detuning, we conduct as many as 50000 realizations. A large portion of the photons undergo a single scattering event. The continuous cooling and trapping is maintained essentially by these single-scattered photons. Photons that are scattered multiple times, on the other hand, can be approximated by a diffusion process and contribute to the atom-atom repulsion described in the main text. The distribution of the number of scattering events is partially summarized in Supplementary Figure 6. As expected, when going into resonance, a higher percentage of photons undergo a larger number of scattering events, thus increasing the effects associated with the diffusive nature of photon transport.

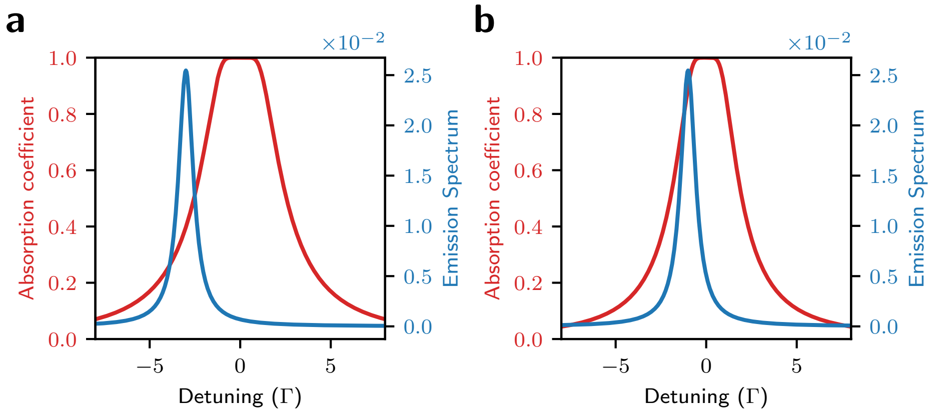

Supplementary Figure 5: Simplified model of photon scattering. Absorption and emission spectra at **a**) -3 Γ and **b**) -1 Γ.

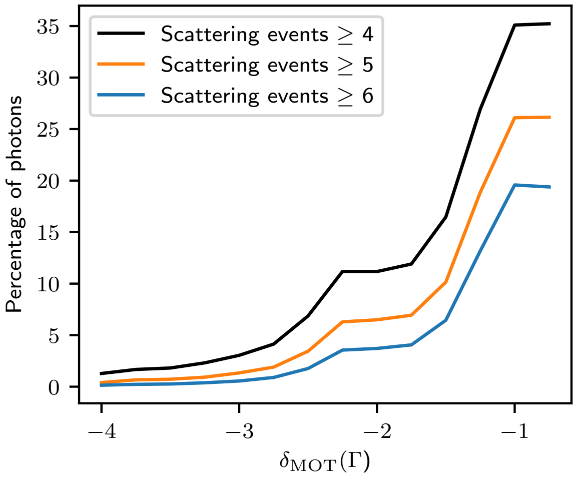

Supplementary Figure 6: Statistics of photon scattering. The plot shows the percentage of photons that undergo more or equal to 4, 5 and 6 scattering events as a function of the MOT main laser detuning.

III. FINITE-SIZE PHOTON BUBBLE MODEL

In this section, we describe simple simulations that allows us to include finite-size effects into the predictions of the homogeneous photon bubble model introduced in the main text. In fact, the typical bubble size is only few times smaller than the typical size of the atom cloud. We thus expect the homogeneous model to start deviating from the experiment already at intermediate length scales, which is in fact observed. These deviations can nonetheless be estimated and, in doing so, construct a finite-size correction to the homogeneous photon bubble model.

As described in the main text, the homogeneous photon bubble model predicts the existence of quasi-coherent structures described by the auto-correlation function:

$$C(r) = j_0(qr)e^{-\gamma r}, \tag{3}$$

where we have restricted ourselves to spherically symmetric solutions, for the same reasons mentioned in the main text. Here, j_0 is the zero-order spherical Bessel function, q essentially the inverse of the bubble size and γ related to photon losses. To simulate the effect of the system's finite size onto the structure defined by Eq. 3 we proceed as follows:

- **1)** – Fix a given value for q and γ.
- **2)** – Generate artificial "density" map by summing a uniform background with white noise. The amplitude of these fluctuations will only determine an overall multiplicative factor in the end, which ultimately will act as a fitting parameter. The size of this artificial density map is ensured to be much larger than $1/q$, in order to approximate an homogeneous system.
- **3)** – The noise is correlated using a filter function. The latter is defined by Eq. 3 together with the parameters set in step 1.

- **4)** – A super-Gaussian envelope of size A is applied, in order to emulate a finite-sized atom cloud.

The procedure above is repeated 100 times. This synthetic dataset is then analysed following the exact same procedure used for the experimental data. All q, γ and A parameters are then fitted to the experimental data. In this way, we are able to include finite-size effects into a model otherwise applicable for very large systems only.

We can also use the procedure above to illustrate finite-size effects – see Supplementary Figure 7. We thus generated synthetic data for two distinct situations, a typical bubble size, $1/q$, much smaller than the total extent of the cloud, A, (left panel); and a second situation where these two parameters are of the same order (right panel). The auto-correlation function of these two hypothetical systems is depicted in red. The dashed black lines depicts a numerical fit of this auto-correlation function with the homogeneous model defined by Eq. 3. While the two are virtually indistinguishable when $1/q \ll A$, noticeable differences occur when the system's size become comparable with the typical bubble length scale. This distortion, however, is systematic and predictable, which allows us to construct an improved photon bubble model.

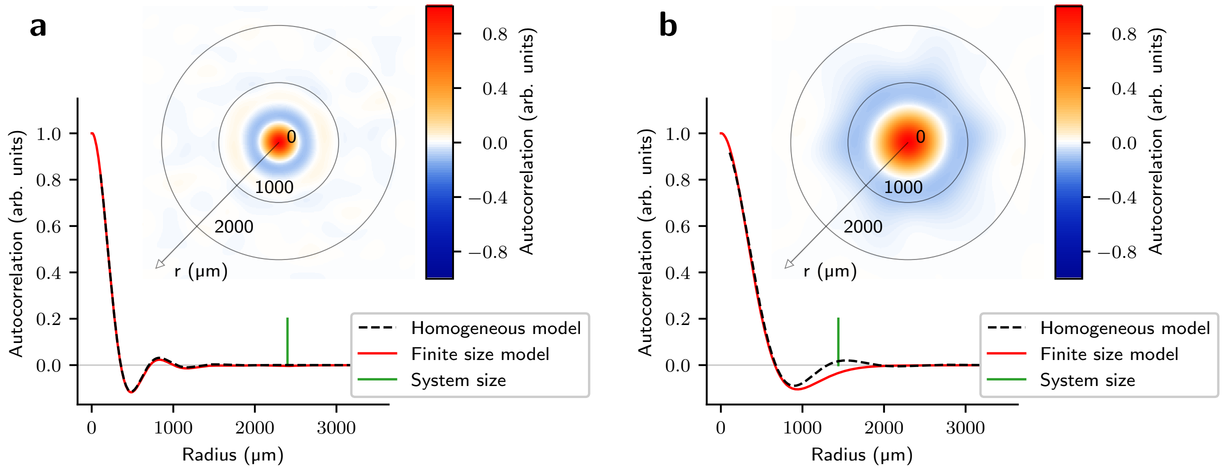

Supplementary Figure 7: Demonstration of finite-size effects. Synthetic dataset considering a system size much larger than the typical bubble length scale – **a** – and a system size of the same order as the bubble length scale – **b**. The red curve represents the radial auto-correlation function calculated for the synthetic data. The dashed black curve is a fit homogeneous model in Eq. 3. The vertical green line indicates the system size, A.

References

[1] Ortiz-Gutiérrez, L. *et al.* Mollow triplet in cold atoms. *New Journal of Physics* **21**, 093019 (2019).

[2] Labeyrie, G. *et al.* Slow diffusion of light in a cold atomic cloud. *Physical review letters* **91**, 223904 (2003).

[3] Labeyrie, G., Müller, C., Wiersma, D., Miniatura, C. & Kaiser, R. Observation of coherent backscattering of light by cold atoms. *Journal of Optics B: Quantum and Semiclassical Optics* **2**, 672 (2000).

[4] Sesko, D. W., Walker, T. & Wieman, C. E. Behavior of neutral atoms in a spontaneous force trap. *JOSA B* **8**, 946–958 (1991).
